# Chicken Consumption and Use of Acid-Suppressing Medications as Risk Factors for *Campylobacter* Enteritis, England

**DOI:** 10.3201/eid1509.080773

**Published:** 2009-09

**Authors:** Clarence C. Tam, Craig D. Higgins, Keith R. Neal, Laura C. Rodrigues, Sally E. Millership, Sarah J. O’Brien, on behalf of the Campylobacter Case–Control Study Group

**Affiliations:** London School of Hygiene and Tropical Medicine, London, UK (C.C. Tam, C.D. Higgins, L.C. Rodrigues); University of Nottingham, Nottingham, UK (K.R. Neal); Essex Health Protection Unit, Witham, UK (S.E. Millership); University of Manchester, Manchester, UK (S.J. O’Brien)

**Keywords:** Campylobacter, infectious intestinal disease, food poisoning, bacteria, gastrointestinal infections, enteric diseases, diarrhea, England, risk factors, research

## Abstract

In a case–control study of *Campylobacter* spp. risk factors in England during 2005–2006, we identified recent consumption of commercially prepared chicken as an important risk factor. The risk for illness associated with recent chicken consumption was much lower for persons who regularly ate chicken than in those who did not, which suggests that partial immunologic protection may follow regular chicken preparation or consumption. Chicken-related risk factors accounted for 41% of cases; acid-suppressing medication, for 10%; self-reported past *Campylobacter* enteritis, 2%; and recent acquisition of a pet dog, 1%. Understanding the risks associated with chicken from different sources will benefit strategies to reduce *Campylobacter* infections. Better characterization of immune correlates for *Campylobacter* infection is necessary to assess the relative importance of immunity and behavioral factors in determining risk.

## Abstract 2

Each of these factors increases risk for *Campylobacter* enteritis.

*Campylobacter* spp. are the most common bacterial cause of enteritis in England. More than 40,000 cases are reported annually (*1*). Incidence of cases reported nationally is ≈80 per 100,000 population, but the community incidence is ≈7× higher (*2*). Previously identified risk factors for *Campylobacter* enteritis include international travel; ingestion of poultry, red meat, unpasteurized milk, and untreated water; contact with pets and farm animals; use of antimicrobial drugs and acid-suppressing medication; and diabetes (*3*–*11*).

Numerous studies implicate chicken consumption as an important risk factor for *Campylobacter* enteritis (*6*–*18*). However, some studies report associations specifically with eating undercooked chicken (*5*,*6*,*12*); others, with any type of chicken; and in 1 study, chicken consumption appeared to be protective (*19*). Other studies have found increased risks only with consumption of commercially prepared chicken (*6*–*8*,*11*,*14*,*20*).

One explanation for these disparities is that studies generally measure the average increase in risk from chicken consumption, without accounting for differences in individual susceptibility. We hypothesized that the frequency of chicken consumption modifies risk for *Campylobacter* enteritis associated with recent chicken consumption, possibly because persons who regularly eat chicken develop partial immunity to *Campylobacter* infection or because they have different consumption or preparation behaviors that influence risk for infection. We report the results of a multicenter case-control study in England designed to investigate food and other risk factors for reported *Campylobacter* enteritis.

## Methods

### Study Participants

Cases were laboratory-confirmed *Campylobacter* spp. infections in persons >18 years of age reported to 1 of 5 English Health Protection Units (HPUs) (East Midlands North, Cheshire and Merseyside, Cumbria and Lancashire, North East and Central London, and Essex) from April 1, 2005, through June 30, 2006. We randomly selected 5 controls per case from records of all persons registered with primary care clinics in the area. Controls were stratum-matched to cases by HPU, age group (18–34, 35–54, and >55 years), sex, and month of report.

Exclusion criteria were international travel in the 14 days before illness for case-patients (or questionnaire completion for controls) and preexisting irritable bowel syndrome. Household clusters were identified by surname and postal address; only the first case in household clusters was included. Controls reporting gastrointestinal symptoms in the preceding 14 days also were excluded.

### Case and Control Recruitment

We recruited case-patients by mail through their local Environmental Health Department or HPU and asked them to return a postage-paid self-completed risk factor questionnaire. We recruited controls by mail through the Health Protection Agency Centre for Infections and asked them to complete a similar questionnaire. Reminders were sent to nonresponders after 2 and 3 weeks. Signed, informed consent was obtained from participants.

### Data Collection

We inquired about demographic information, clinical details, and risk factors in the 5 days before illness for cases and questionnaire completion for controls (i.e., 5-day factors). We also collected information about routine exposures (i.e., habitual factors).

### Statistical Analysis

Risk factors were grouped under 7 domains: health, occupation, pets, water, recreational exposures, food, and household details. We analyzed data by unconditional logistic regression by using Stata 8.2 software (Stata Corporation, College Station, TX, USA). ORs and 95% confidence intervals (CIs) were calculated for each exposure. Analyses were adjusted for age group (18–24, 25–34, 35–44, 45–54, 55–64, and >65 years), sex, study site, and calendar month.

We powered our study to detect an odds ratio (OR) of 1.4 for chicken consumption in the previous week (87% population prevalence (*21*), power = 0.8, α = 0.05), or an OR of 2.4 for an exposure with 1% prevalence.

### Final Multivariable Model

Within each exposure domain, we first constructed a model comprising all habitual exposures. This model was simplified by using backward stepwise elimination until all remaining variables yielded likelihood ratio (LR) test results of p<0.05. This process was repeated for 5-day exposure variables and conducted separately for each exposure domain.

Next, we fitted a model comprising all habitual variables identified in the domain-specific regressions and simplified by backward stepwise elimination as before. A model for all 5-day exposures identified in the domain-specific regressions was similarly constructed.

Lastly, all habitual and 5-day factors from the above regressions were included in 1 model and the final model obtained by backward stepwise elimination. For all risk factors positively associated with disease, we also calculated the proportion of cases attributable to each risk factor (population-attributable fraction).

### Chicken Consumption (Interaction Model)

We investigated further whether regular consumption of chicken modified the risk for disease from recent chicken consumption. We classified participants according to whether they 1) regularly ate chicken (at least once a week) and 2) had eaten it in the previous 5 days. We further classified persons who had eaten chicken in the previous 5 days according to whether they ate it in their own or someone else’s home, at a commercial establishment, or both. We fit a model with an interaction between these variables to investigate how the risk for disease varied in these subgroups relative to persons not exposed to chicken (defined as reporting they did not regularly eat chicken and had not eaten it in the previous 5 days). We assessed statistical evidence for the interaction using the LR test. In a separate model, we additionally adjusted for all other risk factors identified in the multivariable analysis. Because of small numbers in some subgroups, this latter analysis could be performed only for persons who regularly ate chicken and reported eating it in the previous 5 days.

### Sensitivity Analysis

For each of the final multivariable and interaction models, we conducted 2 sensitivity analyses. First, we repeated the analysis excluding case-patients for whom the delay between symptoms onset and questionnaire completion was longer than the median delay for all case-patients. We compared the ORs from this model to those from the model comprising all case-patients to explore potential effects of differential reporting of risk factors among late responders. In the interaction model, we could perform this analysis only for persons who regularly ate chicken and reported eating it in the previous 5 days because of small numbers in other subgroups.

Second, by using an inverse probability-weighted approach (*22*), we investigated whether differences between participants and nonparticipants influenced results. We calculated individuals’ probabilities of participation from a 2-level random intercept logistic model regressing study participation against study site; a 3-way interaction between case and control status, age group, and sex; and area-level deprivation. To account for differences in area-level deprivation, we linked individuals’ postcodes to super output areas (SOAs), geographic boundaries comprising ≈1,000 residents for which aggregated census data are available. SOAs are ranked according to the Index of Multiple Deprivation (*23*), which scores SOAs on 7 domains related to unemployment, income, education, housing, living environment, crime, and healthcare access. We modeled area-level deprivation using SOA as a latent, random intercept variable at the higher level. We then used the inverse probabilities of participation from this model as weights in the final multivariable and interaction models, effectively giving more weight to persons in strata with low participation. We compared the ORs from the weighted and unweighted models to assess potential participation bias.

### Ethical Approval

This study received a favorable ethical opinion from the North West Multicentre Research Ethics Committee. Approval was obtained from local research management and governance departments serving each study site.

## Results

A total of 2,381 (46.5%) case-patients and 5,256 (37.3%) controls returned questionnaires. Participants were excluded for the following reasons: missing age information (2 case-patients, 7 controls); chronic gastrointestinal illness (221 case-patients, 324 controls); gastrointestinal symptoms in the preceding 14 days (431); international travel in the preceding 14 days (560 case-patients, 511 controls); and being part of a household cluster of gastrointestinal illness (6 case-patients). After exclusions, 1,592 cases and 3,983 controls were available for analysis. Among controls, 2,486 (62.4%), 700 (17.6%), and 689 (17.3%) questionnaires were completed after the initial contact, first reminder, and second reminder respectively. Date of questionnaire completion was unknown or implausible for 108 controls.

### Single-Variable Analysis

Habitual factors associated with increased risk were self-reported diarrheal illness in the previous 12 months; self-reported past *Campylobacter* enteritis; use of antimicrobial drugs, antacids and acid-suppressing medications in the previous 28 days; diabetes; puppy ownership; recent dog acquisition; chicken consumption at least once a week; red meat consumption once a week; and sharing of kitchen facilities. Eating commercially prepared chicken in the previous 5 days also was associated with increased risk (Table 1).

**Table 1:** Final multivariable model of both habitual risk factors and risk factors for *Campylobacter* enteritis in the previous 5 days, adjusted for participant age group and sex, study site, and month of year, England, 2005–2006

Exposure domain and variable	Odds ratio	95% Confidence interval	p value
Health details			
Previous *Campylobacter* infection	2.20	1.33–3.64	0.002
Use of acid-suppressing medication in previous 28 days	3.39	2.49–4.62	<0.001
Pets			
Pet fish	0.56	0.33–0.94	0.029
If last pet acquired was a dog, how long ago was it acquired?			
Dog was not last pet bought/no pets	1.00	–	–
>6 months ago	0.76	0.57–1.01	0.057
3–6 months ago	1.30	0.53–3.16	0.566
1–3 months ago	1.74	0.62–4.93	0.296
2–4 weeks ago	14.40	3.69–56.14	<0.001
<2 weeks ago	1.08	0.12–9.90	0.946
Food			
No. times salads eaten per week			
0	1.00	–	–
1	0.89	0.63–1.26	0.503
2	0.58	0.40–0.82	0.002
3	0.72	0.49–1.05	0.086
4	0.93	0.62–1.40	0.739
>5	0.63	0.44–0.91	0.013
No. times legumes eaten per week			
0	1.00	–	–
1	0.65	0.51–0.84	0.001
2	0.57	0.44–0.75	<0.001
3	0.47	0.33–0.68	<0.001
4	0.65	0.40–1.05	0.078
>5	0.66	0.42–1.04	0.071
No. times fruit eaten per week			
0	1.00	–	–
1	0.95	0.53–1.69	0.860
2	1.57	0.96–2.55	0.071
3	1.19	0.71–1.98	0.518
4	1.77	1.05–2.98	0.032
>5	1.06	0.70–1.61	0.775
No. times chicken eaten per week			
0	1.00	–	–
1	1.62	0.98–2.68	0.058
2	1.96	1.16–3.32	0.012
3	1.70	0.98–2.95	0.061
4	2.10	1.16–3.79	0.014
>5	3.74	2.06–6.80	<0.001
Regularly drinks raw milk	1.00	–	–
Rarely/never			
Yes, regularly	0.24	0.08–0.72	0.010
Yes, occasionally	0.70	0.33–1.51	0.365
Location where chicken eaten in past 5 days was prepared			
No chicken eaten	1.00	–	–
In the home/someone else's home only	0.70	0.49–1.00	0.050
Outside the home only	1.95	1.26–3.01	0.003
In the home and outside the home	0.70	0.48–1.03	0.069

Habitual factors associated with decreased risk for illness were vegetarianism; regular consumption of salads, rice, and legumes; occupational exposure to sheep and horses; ownership of fish or rodents; and regular drinking of unpasteurized milk. Consumption of unpasteurized milk and dairy products, noncarbonated and carbonated bottled water, and unfiltered tap water in the previous 5 days also was associated with decreased risk.

### Final Multivariable Model

In the final model, positively associated exposures were past *Campylobacter* enteritis (OR 2.2, 95% CI 1.3–3.6), recent use of acid-suppressing medication (OR 3.4, 95% CI 2.5–4.6), recent acquisition of a dog (OR 14.4, 95% CI 3.7–54.1), regular consumption of chicken (OR 3.7, 95% CI 2.1–6.8 for those eating chicken >5 times a week), and consumption of commercially prepared chicken only in the previous 5 days (OR 2.0, 95% CI 1.3–3.0). Regular consumption of salads, legumes, and unpasteurized milk and consumption of home-prepared chicken in the previous 5 days were associated with decreased risk (Table 2).

**Table 2:** Comparison of increased risks for *Campylobacter* enteritis associated with eating chicken in the previous 5 days in persons who regularly ate and never ate chicken, England, 2005–2006*

Regularly eats chicken	Ate chicken in previous 5 days	Location where chicken was prepared	OR†	95% CI	p value
No	No	–	1.00	–	–
Yes	Yes	In the home only	1.47	0.96–2.26	0.078
Yes	Yes	Outside the home only	3.86	2.33–6.39	<0.001
Yes	Yes	Inside the home, and prepared outside the home	1.59	1.02–2.47	0.042

*OR, odds ratio; CI, confidence interval. †ORs adjusted for participant age group and sex; study site; study month; use of acid suppressing medication; self-reported past *Campylobacter* enteritis; recent acquisition of a dog; and frequency of consuming of salads, fruit, vegetables, and unpasteurized milk.

### Chicken Consumption (Interaction Model)

Statistical evidence was strong for an interaction between regular and recent chicken consumption (LR test p = 0.0002) (Figure). Overall, persons who regularly ate chicken (at least once a week) were at greater risk for illness than those who did not (OR 1.6, 95% CI 1.2–2.0) (Figure, top). However, for persons who did not regularly eat chicken, eating it in the previous 5 days posed a 5-fold greater risk than it did for persons who did not (OR 5.0, 95% CI 2.1–11.9). We did not see this association for persons who regularly ate chicken (OR 0.8, 95% CI 0.6–1.0) (Figure, middle).

**Figure:**
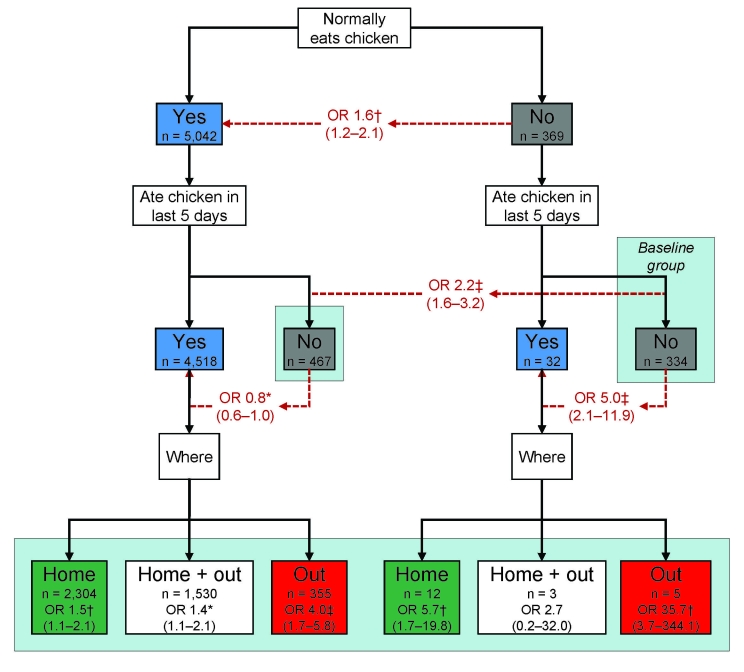
Odds ratios (ORs) and 95% confidence intervals (shown in parentheses) for *Campylobacter* enteritis associated with chicken consumption, England, 2005–2006. Numbers in boxes represent persons in each category; Numbers in red indicate relevant comparisons; arrows indicate direction of risk. For boxes in the bottom level, ORs compare risk for *Campylobacter* enteritis between individuals in that group and the baseline group (labeled), which comprises persons who do not regularly eat chicken and did not eat chicken in the previous 5 days (n = 334). Model is adjusted for age group, sex, study site, and month. *p<0.05; †p<0.01; ‡p<0.001.

The risk associated with eating commercially prepared chicken was greater than that associated with eating home-prepared chicken. Among persons who regularly ate chicken, eating commercially prepared chicken in the previous 5 days was associated with a 4-fold increased risk (OR 4.0, 95% CI 2.8–5.8) for *Campylobacter* infection, much higher than the risk associated with eating home-prepared chicken (OR 1.5, 95% CI 1.1–2.1). Among those who did not regularly eat chicken, eating commercially prepared chicken was associated with a 36-fold increased risk (OR 35.7, 95% CI 3.7–344.1); however, this group was very small (Figure, bottom).

Adjusting for nonchicken-related factors had little effect on the ORs (Table 2). The p values were considerably higher, although this analysis was based on fewer persons because of missing data in some variables.

### Sensitivity Analyses

Excluding late-responding case-patients had little effect on the ORs in either the final multivariable model or the interaction model. In the final multivariable model, ORs for eating chicken >1 times per week were consistently higher than in the model comprising all cases (OR 5.4, 95% CI 2.3–12.4 for eating chicken >5 times per week).

In the inverse probability-weighted final model, the OR for eating commercially prepared chicken in the previous 5 days was 1.6 (95% CI 0.98–2.62). Other results did not change. In the interaction model, the weighted model indicated stronger evidence for associations with eating home-prepared chicken (OR 1.76, 95% CI 1.22–2.29, p = 0.001) and eating home-prepared and commercially prepared chicken in the previous 5 days (OR 1.63, 95% CI 1.18–2.25, p = 0.003), compared with the unweighted results (Table 1).

### Population-Attributable Fractions

Chicken-related exposures were reported by 92.5% of controls; use of acid-suppressing medications, by 6.0%; past *Campylobacter* enteritis, by 2.2%; and recent acquisition of a dog, by 1.6%. The percentage of cases attributable to each of these risk factors (Table 3) was as follows: chicken-related exposures, 41%; acid-suppressing medications, 10%; past *Campylobacter* enteritis, 3%; and recent acquisition of a dog, 1%.

**Table 3:** Population–attributable fractions for identified risk factors for *Campylobacter* enteritis, England, 2005–2006*

Variable	PAF, %	SE	95% CI
Previous *Campylobacter* infection	2.6	1.37	0.0–5.3
Proton pump inhibitor use in previous 28 days	10.4	1.70	7.0–13.6
Acquisition of dog in previous month	1.2	1.10	0.0–3.3
Chicken consumption	40.6	11.84	12.2–59.8

*PAF, population–attributable fraction; CI, confidence interval.

## Discussion

Chicken consumption and use of acid-suppressing medications are major risk factors for *Campylobacter* enteritis in England. Chicken-related exposures accounted for 41% of adult cases, consistent with previous US and Australian studies (*5*,*8*,*24*). Recent use of acid-suppressing medications increased risk for illness 3-fold, similar to other studies (*9*), accounting for 10% of cases.

Like others (*3*,*6*–*8*,*11*), we found that commercially prepared chicken poses a greater risk than home-prepared chicken. Reasons might be greater contamination levels or inadequate cooking procedures, which could be more common in commercial establishments than in homes. However, we found only modest increases in risk for persons who ate home-prepared and commercially prepared chicken, suggesting that persons who regularly eat chicken at home frequent different types of establishments than do persons who tend to eat chicken only outside the home. We could not investigate this hypothesis further.

Several findings suggest that acquired immunity might be important. The risk for *Campylobacter* enteritis associated with recent chicken consumption depended on whether participants regularly ate chicken. For persons who ate chicken in the previous 5 days, the risk was considerably greater for those who did not regularly eat chicken than for those who did. Recent, but not longer-term, dog owners had higher risk for illness, whereas persons who regularly drank unpasteurized milk had decreased risk. We could not confirm participants’ immunologic status; however, these results suggest that long-term exposure to these sources of *Campylobacter* spp. might confer partial immunity (*25*). In immunologically susceptible populations, however, unpasteurized milk is a well-known cause of outbreaks of infection with *Campylobacter* and potentially fatal Shiga–toxin producing *Escherichia coli* (*26*). Further developments to characterize relevant correlates of immune status for *Campylobacter* infection are required to confirm these findings.

Despite the potential role of immunity, participants reporting previous *Campylobacter* enteritis, but not nonspecific enteritis, had greater risk for recent *Campylobacte*r illness than did persons not reporting past *Campylobacter* enteritis. Compared with all cases, those reporting a previous episode of *Campylobacter* enteritis were of similar age but more likely to be female (57% vs. 49%). These persons may differ in other ways that increase risk, such as medical history or immune competence. However, this finding should be interpreted cautiously because we had no independent confirmation of self-reported *Campylobacter* enteritis.

Other researchers (*11*) have suggested that regular consumption of vegetables and legumes might protect against infection. However, eating these foods might simply be a marker for unmeasured behavior related to decreased risk.

We found no associations with any environmental variables. Environmental exposures may pose low or transient risk; temporal variation in environmental prevalence of *Campylobacter* spp. could make their effects difficult to detect. Previous studies in England have identified diabetes as a risk factor for *Campylobacter* enteritis (*9*); in our study, initial analyses suggested a 1.5-fold increase in illness associated with diabetes, but this effect disappeared after adjustment for other habitual factors.

We did not include persons who reported recent international travel because travel-related illness may have different risk factors. However, international travel is common among persons in England with laboratory-confirmed *Campylobacter* infection; 24% of all case-patients reported traveling abroad in the previous 14 days compared with 11% of controls.

Our analysis emphasizes the importance of accounting for regular dietary habits in determining risk associated with recent consumption of putatively risky foods. Moreover, selection of an appropriate baseline comparison group (in this case, persons truly unexposed to chicken consumption) is crucial to enable meaningful comparisons. In the future, distinguishing long-term and recent exposures will be important in investigating how their association influences risk. More detailed study of the risks associated with chicken prepared at home and in commercial establishments is needed. Given the limitations of case–control studies for collecting long-term exposure information, innovative studies using a variety of approaches are necessary.

In England, chicken consumption is the major recognized risk factor for *Campylobacter* enteritis. Understanding the differing risks from poultry sources should guide strategies to reduce risk for infection from chicken. Immunologic factors appear to be important in determining risk for *Campylobacter* enteritis given exposure to infection. Meaningful interpretation of *Campylobacter* risk factor studies requires better knowledge of population susceptibility to infection and the extent to which past exposure can induce protection. Identifying relevant immune correlates would help determine whether differences in immune status, behavior, or both are responsible for differing risks for *Campylobacter* enteritis between populations or population subgroups.
